# Impact of stent retriever diameter on clinical outcomes in mechanical thrombectomy: a multicenter comparison of the 4-mm and 6-mm Solitaire devices

**DOI:** 10.3389/fneur.2025.1618737

**Published:** 2025-07-09

**Authors:** Jung Hyun Park, Hoon Kim, Sunghan Kim

**Affiliations:** ^1^Department of Neurosurgery, Dongtan Sacred Heart Hospital, Hallym University College of Medicine, Hwaseong, Republic of Korea; ^2^Department of Neurosurgery, Bucheon St. Mary’s Hospital, College of Medicine, The Catholic University of Korea, Seoul, Republic of Korea

**Keywords:** cerebral infarction, thrombectomy, stents, equipment design, treatment outcome

## Abstract

**Objective:**

Optimal stent retriever diameter for mechanical thrombectomy in large vessel occlusion remains debated. We compared the efficacy and safety of Solitaire 4 × 40 mm and 6 × 40 mm stent retrievers in treating distal internal carotid artery (ICA) or middle cerebral artery (MCA) M1 segment occlusion.

**Methods:**

We retrospectively analyzed 145 patients with distal ICA or M1 occlusion treated with the Solitaire 4 × 40 mm (*n* = 59) or 6 × 40 mm (*n* = 86) devices. The primary outcome was the rate of first-pass modified thrombolysis in cerebral infarction (mTICI) ≥ 2b without rescue therapy. Secondary outcomes included the number of passes to achieve mTICI ≥2b, device switching and distal embolization rates, and safety parameters.

**Results:**

The 6 × 40 mm group displayed a significantly higher first-pass mTICI ≥2b rate (58.1% vs. 23.7%, *p* < 0.001), a lower number of passes to achieve successful revascularization (1.67 ± 1.00 vs. 2.54 ± 1.19, *p* < 0.001), and lower rates of device switching (4.7% vs. 18.6%, *p* = 0.015) and distal embolization (8.1% vs. 25.4%, *p* = 0.009). Postprocedural hemorrhage was less frequent in the 6 × 40 mm group (55.8% vs. 76.3%, *p* = 0.039), whereas symptomatic hemorrhage rates were similar between the groups (5.8% vs. 5.1%, *p* > 0.999).

**Conclusion:**

The Solitaire 6 × 40 mm stent retriever showed more favorable efficacy outcomes and comparable safety compared to the 4 × 40 mm device for distal ICA or M1 occlusion, suggesting it may be considered a preferred option for these vessels.

## 1 Introduction

Mechanical thrombectomy is the standard of care for acute ischemic stroke caused by large vessel occlusion, with stent retrievers playing a pivotal role in achieving successful revascularization. The continuous evolution of these devices has led to various modifications in their design, including length, diameter, and strut configuration, all of which can potentially influence treatment outcomes (1, 2). Understanding the effects of these device specifications is crucial for optimizing procedural success and patient outcomes.

Device length in mechanical thrombectomy has been extensively studied, with longer devices generally associated with improved outcomes (3–5). By contrast, the impact of stent retriever diameter—another key mechanical characteristic of stent retrievers—remains poorly understood because of limited and conflicting evidence (3, 6–8). Although some studies suggested that larger diameters enhance efficacy (8), others highlighted potential drawbacks (7), contributing to ongoing uncertainty in device selection. This inconsistency is largely attributable to heterogeneous study designs that failed to control for critical variables, such as device length and manufacturer differences, introducing variability that complicates interpretation.

To address these limitations, the present study specifically compared stent retrievers with different diameters but the same device length. By isolating diameter as the primary variable, this study examined its role in influencing clinical and angiographic outcomes in mechanical thrombectomy. We hypothesized that differences in the stent retriever diameter significantly affect procedural efficacy and safety, particularly in terms of first-pass success and complication rates.

## 2 Methods

### 2.1 Study design and patient characteristics

This study retrospectively analyzed prospectively collected data from patients who underwent mechanical thrombectomy for acute ischemic stroke at two stroke centers in South Korea between January 2019 and November 2021. The study population included patients with symptomatic large vessel occlusion in the anterior circulation, particularly involving the distal internal carotid artery (ICA) or the M1 segment of the middle cerebral artery (MCA). All patients were treated using the Solitaire 4 × 40 mm or 6 × 40 mm stent retriever (Medtronic, Irvine, CA, USA).

**Figure 1:**
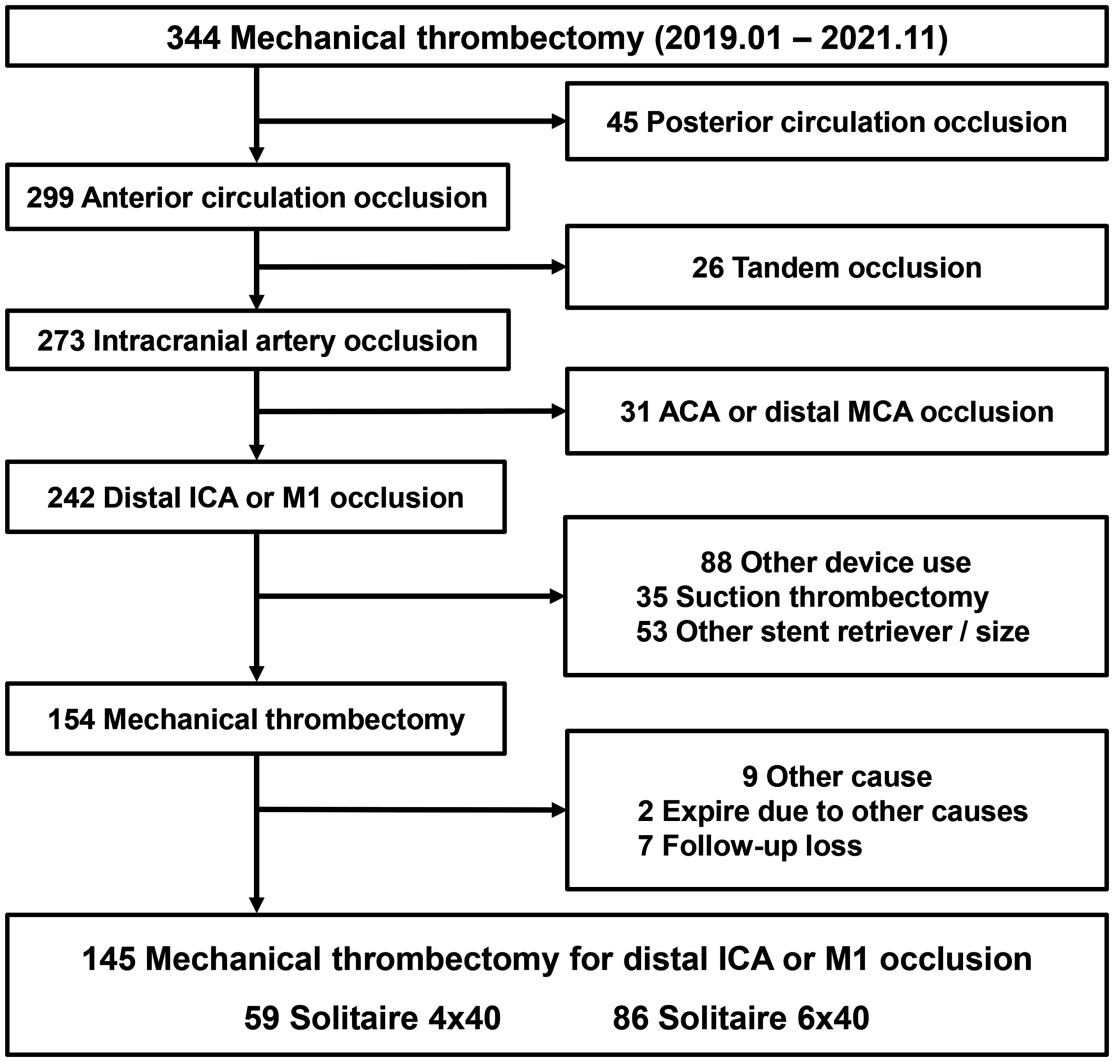
Flow chart of patient selection comparing Solitaire 6 × 40 mm vs. 4 × 40 mm in anterior circulation large artery occlusion.

The inclusion criteria for this study were as follows: (1) patients of any age with symptomatic occlusion of the distal ICA or the M1 segment of the MCA; (2) <24 h from stroke symptom onset to femoral puncture, with patients within 6 h included based on time criteria and those between 6–24 h based on imaging evidence of salvageable tissue such as diffusion/perfusion mismatch; (3) Alberta Stroke Program Early CT Score ≥6 at presentation; (4) pretreatment National Institutes of Health Stroke Scale (NIHSS) score ≥6; and (5) premorbid modified Rankin scale (mRS) ≤ 1. The exclusion criteria were as follows: (1) posterior circulation occlusions; (2) tandem occlusions; (3) anterior circulation occlusions outside the distal ICA or M1 (e.g., occlusions in the anterior cerebral artery or more distal branches of the MCA, such as M2 and M3); (4) mechanical thrombectomy performed with aspiration alone; (5) use of Solitaire devices other than the 4 × 40 mm or 6 × 40 mm sizes or any other stent retrievers; and (6) patients with missing outcome data due to death from unrelated causes or loss to follow-up. The inclusion and exclusion criteria applied for patient selection are outlined in Figure 1.

After applying the exclusion criteria, 145 patients were included. They were categorized into two groups based on the size of the Solitaire device used: 59 patients in the Solitaire 4 × 40 mm group and 86 patients in the Solitaire 6 × 40 mm group. Clinical and angiographic data were obtained from patients’ electronic medical records and a prospectively maintained mechanical thrombectomy database. This study was approved by our institutional review board, and the requirement for informed consent was waived (subject number: HC22RIDI0104).

### 2.2 Mechanical thrombectomy technique and device selection

All mechanical thrombectomy procedures were performed under local anesthesia using femoral artery access with an 8F sheath. A balloon guide catheter was utilized in most cases. The selection of the Solitaire 4 × 40 mm or 6 × 40 mm stent retriever was primarily based on vascular compatibility with the occluded artery. The 4-mm device was generally preferred for M1 segment of the MCA occlusions, while the 6-mm device was more commonly used for distal ICA occlusions. Additionally, in cases of long-segment occlusions with a high thrombus burden, the 6-mm device was prioritized to facilitate clot retrieval. Ultimately, the final choice of device was based on the operator’s discretion. In all cases, a combined suction technique (Solumbra technique) using a distal catheter was applied. If recanalization was not achieved despite sufficient attempts with the initial device, rescue therapy with intra-arterial thrombolysis or angioplasty/stenting was performed as deemed necessary by the operator.

### 2.3 Assessment of efficacy and safety outcomes

The primary efficacy outcomes were assessed using angiographic and clinical parameters. For angiographic outcomes, we evaluated reperfusion time (time from stroke onset to reperfusion), procedure time, number of device passes, and the necessity for device switching or rescue device use. The first-pass effect was defined as achieving a modified thrombolysis in cerebral infarction (mTICI) score of ≥2b or 3 after the initial pass, and final mTICI scores were recorded. We also documented emboli to new territory and reocclusion rates as potential complications. Clinical efficacy was primarily assessed using the mRS at 3 months post-procedure.

The primary safety outcomes included procedure-related complications (vessel perforation, dissection, and vasospasm) and postprocedural hemorrhage. Hemorrhage was classified according to the European Cooperative Acute Stroke Study criteria as hemorrhagic infarction (HI-1 and HI-2) and parenchymal hematoma (PH-1 and PH-2). The incidence of symptomatic hemorrhage was also recorded.

To identify predictors of first-pass recanalization, univariate and multivariate logistic regression analyses were performed. Additionally, we performed subgroup analyses of patients with embolic infarction and those with M1 occlusion, evaluating both efficacy and safety outcomes in these specific populations.

### 2.4 Statistical analysis

Continuous variables were described as the mean ± standard deviation and analyzed using Student’s *t*-test. Normality was assessed using the Shapiro–Wilk test, and all variables met the assumption for parametric testing. Categorical variables were summarized as frequencies and percentages (%) and analyzed using Pearson’s χ^2^ test or Fisher’s exact test.

To determine the predictors of first-pass recanalization, univariate logistic regression analysis was initially performed. The adjusted variables included stent retriever diameter, patient demographics (age and sex), medical history (atrial fibrillation, hypertension, diabetes mellitus, dyslipidemia, coronary artery disease, previous ischemic stroke, and smoking status), initial NIHSS score, use of intravenous tissue plasminogen activator, stroke subtype, vessel treated on first pass, time from stroke onset to puncture, time from stroke onset to reperfusion, and procedure time. Variables showing significant associations (*p* < 0.05) in univariate analysis were initially included in the primary multivariable logistic regression model (multivariable analysis 1). To explore whether a more inclusive threshold might identify additional relevant predictors, a secondary multivariable logistic regression analysis (multivariable analysis 2) was conducted using a relaxed inclusion criterion of *p* < 0.10 in the univariate analysis. The results of logistic regression analyses were reported as odds ratios (ORs) with two-sided 95% confidence intervals (CIs). All statistical tests were two-sided, with *p* < 0.05 indicating significance. All statistical analyses were performed using R software (version 4.2.1; R Foundation for Statistical Computing, Vienna, Austria) and cross-validated using Python (version 3.11; Python Software Foundation) for reproducibility.

## 3 Results

### 3.1 Baseline characteristics

The baseline characteristics of patients treated with the Solitaire 4 × 40 mm and 6 × 40 mm stent retrievers were compared (Table 1). Age, sex distribution, and the prevalence of risk factors did not significantly differ between the groups. Laboratory results were largely similar between the groups, with the exception of a significantly higher platelet count in the Solitaire 4 × 40 mm group (241.71 ± 90.76/mm^3^ vs. 213.60 ± 64.20/mm^3^, *p =* 0.031).

**Table 1:** Comparison of baseline characteristics.

Variables	Solitaire 4 × 40 mm (*n =* 59)	Solitaire 6 × 40 mm (*n =* 86)	*p*	Reference value
Age (years)	70.78 ± 13.94	70.24 ± 14.27	0.823	
Female sex	24 (40.7)	34 (39.5)	> 0.999	
Risk factors				
Atrial flutter or fibrillation	13 (22.0)	29 (33.7)	0.181	
Hypertension	35 (59.3)	54 (62.8)	0.804	
Diabetes mellitus	12 (20.3)	18 (20.9)	> 0.999	
Dyslipidemia	8 (13.6)	24 (27.9)	0.065	
Coronary artery disease	5 (8.5)	13 (15.1)	0.35	
Ischemic stroke	8 (13.6)	14 (16.3)	0.831	
Smoking	10 (16.9)	15 (17.4)	> 0.999	
Laboratory results				
Hemoglobin	14.16 ± 2.06	13.56 ± 1.96	0.077	13.0–18.0 g/dL
White blood cell count	1803.93 ± 3744.38	3135.82 ± 4621.97	0.068	4.0–10.0/mm^3^
Platelet count	241.71 ± 90.76	213.60 ± 64.20	0.031	140–450/mm^3^
BUN	20.24 ± 19.57	22.18 ± 27.59	0.643	6.0–20.0 mg/dL
Creatinine	0.99 ± 0.45	1.04 ± 0.72	0.619	0.70–1.20 mg/dL
Initial NIHSS	17.61 ± 6.05	16.59 ± 5.73	0.306	
IV tPA	26 (44.1)	28 (32.6)	0.217	
Stroke onset to puncture (min)	269.0 ± 264.02	255.35 ± 196.87	0.722	
Primary occlusion site			< 0.001	
ICA	5 (8.5)	36 (41.9)		
MCA-M1	54 (91.5)	50 (58.1)		
Stroke subtype			0.109	
Embolic infarction	50 (84.7)	81 (94.2)		
Atherosclerosis	9 (15.3)	5 (5.8)		
Balloon guiding catheter	58 (98.3)	86 (100.0)	0.406^*^	
Distal catheter	59 (100.0)	86 (100.0)	N/A	

Values are presented as the mean ± standard deviation or number (%) as otherwise indicated. Hb, hemoglobin; BUN, blood urea nitrogen; NIHSS, National Institute of Health Stroke Scale; IV tPA, intravenous tissue plasminogen activator; ICA, internal carotid artery; MCA-M1, M1 segment of the middle cerebral artery. *p value calculated using Fisher’s exact test.

The initial NIHSS scores (17.61 ± 6.05 vs. 16.59 ± 5.73, *p =* 0.306) and the proportion of patients receiving IV thrombolysis (44.1% vs. 32.6%, *p =* 0.217) did not differ between the groups. The time from stroke onset to puncture was also similar between the groups (269.0 ± 264.02 min vs. 255.35 ± 196.87 min, *p =* 0.722).

A notable difference was observed in the primary occlusion site distribution (*p <* 0.001). Particularly, the Solitaire 6 × 40 mm group had a higher proportion of ICA occlusions (41.9% vs. 8.5%), whereas the Solitaire 4 × 40 mm group had a higher rate of M1 segment MCA occlusions (91.5% vs. 58.1%). Embolic infarctions were the predominant stroke subtype in both groups (94.2% vs. 84.7%). The use of balloon guiding catheters was nearly universal in both groups (98.3% vs. 100%), and all procedures were performed using distal catheters.

### 3.2 Clinical and angiographic outcomes by stent retriever diameter

Clinical and angiographic outcomes were compared between the Solitaire 4 × 40 mm and 6 × 40 mm stent retrievers (Table 2). The time from stroke onset to reperfusion and the procedure time were not significantly different between the groups. The Solitaire 6 × 40 mm group exhibited superior first-pass efficacy, including a significantly higher rate of first-pass mTICI ≥2b (58.1% vs. 23.7%, *p <* 0.001) and first-pass mTICI 3 (87.2% vs. 66.1%, *p =* 0.005). Consequently, the Solitaire 6 × 40 mm group required fewer device passes (1.67 ± 1.00 vs. 2.54 ± 1.19, *p <* 0.001). The final angiographic outcomes also favored the Solitaire 6 × 40 mm group, which had a higher rate of mTICI 3 (87.2% vs. 66.1%, *p =* 0.007). The overall rate of successful reperfusion (final mTICI ≥2b) was slightly higher in the Solitaire 6 × 40 mm group (98.8% vs. 94.9%, *p =* 0.304).

**Table 2:** Clinical and angiographic outcomes by stent retriever diameter.

Variables	Solitaire 4 × 40 mm (*n =* 59)	Solitaire 6 × 40 mm (*n =* 86)	*p*
Stroke onset to reperfusion (min)	301.76 ± 267.15	286.73 ± 198.86	0.698
Procedure time (min)	32.76 ± 18.77	31.78 ± 25.20	0.799
First-pass mTICI ≥2b	14 (23.7)	50 (58.1)	< 0.001
First-pass mTICI 3	39 (66.1)	75 (87.2)	0.005
No. of device passes	2.54 ± 1.19	1.67 ± 1.00	< 0.001
> 3 passes	8 (13.6)	4 (4.7)	0.069^*^
Angiographic outcome			
Final mTICI			0.007^*^
3	39 (66.1)	75 (87.2)	
2b	17 (28.8)	10 (11.6)	
2a	1 (1.7)	1 (1.2)	
1	1 (1.7)	0 (0.0)	
0	1 (1.7)	0 (0.0)	
Final mTICI ≥2b	56 (94.9)	85 (98.8)	0.304^*^
Emboli to new territory	15 (25.4)	7 (8.1)	0.009
Device switch	11 (18.6)	4 (4.7)	0.015
Rescue device used	6 (10.2)	2 (2.3)	0.063^*^
Reocclusion	3 (5.1)	6 (7.0)	0.739^*^
Three-month mRS	2.36 ± 1.09	2.24 ± 1.22	0.620^*^
0	2 (3.4)	7 (8.1)	
1	12 (20.3)	15 (17.4)	
2	18 (30.5)	31 (36.0)	
3	17 (28.8)	18 (20.9)	
4	10 (16.9)	13 (15.1)	
5	0 (0.0)	2 (2.3)	

Values are presented as the mean ± standard deviation or number (%) as otherwise indicated. mTICI, modified treatment in cerebral ischemia; mRS, modified Rankin scale. *p value calculated using Fisher’s exact test.

Notably, the Solitaire 4 × 40 mm group had a higher rate of embolization to new territory (25.4% vs. 8.1%, *p =* 0.009) and a higher device switch rate (18.6% vs. 4.7%, *p =* 0.015). The use of rescue devices was also more common in the Solitaire 4 × 40 mm group, although this difference was not statistically significant. Regarding clinical outcomes, the mean 3-month mRS was similar between the two groups (2.36 ± 1.09 vs. 2.24 ± 1.22, *p =* 0.620).

### 3.3 Safety outcomes by stent retriever diameter

Safety outcomes were compared between the Solitaire 4 × 40 mm and 6 × 40 mm stent retriever groups (Table 3). Both groups demonstrated overall favorable safety profiles with low rates of procedural complications. Vessel perforation occurred in one patient (1.2%) in the Solitaire 6 × 40 mm group, versus none in the Solitaire 4 × 40 mm group. Dissection rates (1.7% vs. 2.3%, *p* > 0.999) and reocclusion rates (5.1% vs. 7.0%, *p =* 0.739) were comparable between the groups. However, the incidence of vasospasm was significantly higher in the Solitaire 4 × 40 mm group (8.5% vs. 1.2%, *p =* 0.04).

**Table 3:** Safety outcomes by stent retriever diameters.

Variables	Solitaire 4 × 40 mm (*n =* 59)	Solitaire 6 × 40 mm (*n =* 86)	*p*
Vessel perforation	0 (0.0)	1 (1.2)	> 0.999^*^
Dissection	1 (1.7)	2 (2.3)	> 0.999^*^
Vasospasm	5 (8.5)	1 (1.2)	0.04^*^
Reocclusion	3 (5.1)	6 (7.0)	0.739^*^
^†^Postprocedural hemorrhage	45 (76.3)	48 (55.8)	0.039^*^
H-1	30 (50.8)	34 (39.5)	
H-2	12 (20.3)	7 (8.1)	
PH-1	2 (3.4)	4 (4.7)	
PH-2	1 (1.7)	3 (3.5)	
Symptomatic hemorrhage	3 (5.1)	5 (5.8)	> 0.999^*^

Values are presented as the mean ± standard deviation or number (%) as otherwise indicated. *p-value calculated using Fisher’s exact test. †Classified according to the European Cooperative Acute Stroke Study criteria. H, hemorrhagic infarction, PH, parenchymal hematoma.

Postprocedural hemorrhage was more common in the Solitaire 4 × 40 mm group (76.3% vs. 55.8%, *p =* 0.039), although most hemorrhages were minor (H-1 and H-2). The rates of more severe parenchymal hemorrhage (PH-1 and PH-2) were comparable between the groups, as was the rate of symptomatic hemorrhage (5.1% vs. 5.8%, *p* > 0.999).

### 3.4 Predictors of achieving first-pass recanalization

Predictors of first-pass recanalization were examined using logistic regression analyses (Table 4). In univariate analysis, the use of the Solitaire 6 × 40 mm stent retriever was significantly associated with achieving first-pass recanalization (OR = 5.44, 95% CI = 2.53–11.69, *p <* 0.001). Additionally, lower initial NIHSS scores (OR = 0.93, 95% CI = 0.87–0.98, *p =* 0.011) and shorter procedure times (OR = 0.97, 95% CI = 0.95–0.99, *p =* 0.002) were also associated with first-pass recanalization.

**Table 4:** Predictor of achieving first-pass recanalization.

Variables	Univariate analysis		Multivariable analysis 1^†^		Multivariable analysis 2^‡^	
	OR (95% CI)	*p*	OR (95% CI)	*p*	OR (95% CI)	*p*
Large stent retriever diameter (Solitaire 6 × 40 mm)	5.44 (2.53–11.69)	<0.001	6.03 (2.6–14.01)	<0.001	6.14 (2.58–14.64)	<0.001
Age	1.0 (0.97–1.02)	0.778				
Female sex	1.15 (0.59–2.25)	0.681				
Atrial flutter or fibrillation	0.52 (0.24–1.12)	0.094			0.58 (0.23–1.5)	0.261
Hypertension	0.78 (0.4–1.54)	0.479				
Diabetes mellitus	1.22 (0.54–2.74)	0.627				
Dyslipidemia	0.89 (0.4–1.98)	0.782				
Coronary artery disease	2.34 (0.85–6.44)	0.099			1.55 (0.43–5.62)	0.503
Ischemic stroke	0.91 (0.36–2.3)	0.849				
Smoking	2.33 (0.97–5.62)	0.060			1.78 (0.62–5.16)	0.286
Initial NIHSS	0.93 (0.87–0.98)	0.011	0.88 (0.81–0.95)	0.001	0.88 (0.82–0.96)	0.003
IV tPA	0.88 (0.44–1.73)	0.705				
Stroke subtype	0.33 (0.09–1.25)	0.103				
Vessel treated on first pass	1.43 (0.68–3.01)	0.347				
Stroke onset to puncture (min)	1.0 (1.0–1.0)	0.146				
Stroke onset to reperfusion (min)	1.0 (1.0–1.0)	0.261				
Procedure time (min)	0.97 (0.95–0.99)	0.002	0.95 (0.93–0.98)	<0.001	0.96 (0.93–0.98)	0.001

NIHSS, National Institute of Health Stroke Scale; IV tPA, intravenous tissue plasminogen activator.

† Multivariable analysis 1: adjusted for variables with *p* < 0.05 in univariate analysis.

‡ Multivariable analysis 2: adjusted for variables with *p* < 0.1 in univariate analysis.

In multivariable analysis 1, which included variables with *p < 0.05* from univariate analysis, the use of the Solitaire 6 × 40 mm stent retriever remained an independent predictor of first-pass recanalization (OR = 6.03, 95% CI = 2.60–14.01, *p <* 0.001). Similarly, both lower NIHSS scores (OR = 0.88, 95% CI = 0.81–0.95, *p =* 0.001) and shorter procedure times (OR = 0.95, 95% CI = 0.93–0.98, *p <* 0.001) remained significant predictors of first-pass success.

In multivariable analysis 2, which incorporated variables with *p <* 0.10 from univariate analysis, the use of the Solitaire 6 × 40 mm stent retriever remained a robust independent predictor of first-pass recanalization (OR = 6.14, 95% CI = 2.58–14.64, *p <* 0.001). Lower initial NIHSS scores (OR = 0.88, 95% CI = 0.82–0.96, *p =* 0.003) and shorter procedure times (OR = 0.96, 95% CI = 0.93–0.98, *p =* 0.001) were also independently associated with successful first-pass recanalization. Although several additional variables such as atrial flutter or fibrillation (OR = 0.58, 95% CI = 0.23–1.50, *p =* 0.261), coronary artery disease (OR = 1.55, 95% CI = 0.43–5.62, *p =* 0.503), and smoking (OR = 1.78, 95% CI = 0.62–5.16, *p =* 0.286) were included in this model based on the relaxed inclusion threshold, none demonstrated statistically significant associations.

### 3.5 Subgroup analysis of clinical and safety outcomes in embolic infarction

A subgroup analysis of patients with embolic infarctions was conducted to compare efficacy and safety outcomes between the Solitaire 4 × 40 mm and 6 × 40 mm stent retriever groups (Tables 5, 6). The time from stroke onset to reperfusion and procedure time did not differ between the groups. Consistent with the overall cohort, the Solitaire 6 × 40 mm group demonstrated superior first-pass efficacy in embolic infarctions, with significantly higher first-pass mTICI ≥2b (59.3% vs. 24.0%, *p <* 0.001) and first-pass mTICI 3 rates (88.9% vs. 72.0%, *p =* 0.026). The Solitaire 6 × 40 mm group required fewer device passes (1.65 ± 1.00 vs. 2.46 ± 1.11, *p <* 0.001). The final angiographic outcomes also favored the Solitaire 6 × 40 mm group, which had a higher mTICI 3 rate (88.9% vs. 72.0%, *p =* 0.026). Notably, the overall rate of successful reperfusion (final mTICI ≥2b) was 100% in both groups.

**Table 5:** Subgroup analysis of clinical and angiographic outcomes in embolic infarction by stent retriever diameter.

Variables	Solitaire 4 × 40 mm (*n =* 50)	Solitaire 6 × 40 mm (*n =* 81)	*p*
Stroke onset to reperfusion (min)	290.30 ± 244.47	276.99 ± 199.12	0.734
Procedure time (min)	28.64 ± 14.80	31.30 ± 25.71	0.507
First-pass mTICI ≥ 2b	12 (24.0)	48 (59.3)	<0.001
First-pass mTICI 3	36 (72.0)	72 (88.9)	0.026
No. of device passes	2.46 ± 1.11	1.65 ± 1.00	<0.001
>3 passes	6 (12.0)	4 (4.9)	0.179^*^
Angiographic outcome			
Final mTICI			0.026
3	36 (72.0)	72 (88.9)	
2b	14 (28.0)	9 (11.1)	
2a	0 (0.0)	0 (0.0)	
1	0 (0.0)	0 (0.0)	
0	0 (0.0)	0 (0.0)	
Final mTICI ≥ 2b	50 (100.0)	81 (100.0)	NA
Emboli to new territory	13 (26.0)	6 (7.4)	0.007
Device switch	10 (20.0)	4 (4.9)	0.016
Rescue device used	0 (0.0)	0 (0.0)	NA
Reocclusion	0 (0.0)	3 (3.7)	0.287^*^
3-month mRS	2.32 ± 1.08	2.20 ± 1.20	0.637^*^
0	2 (4.0)	7 (8.6)	
1	10 (20.0)	14 (17.3)	
2	15 (30.0)	30 (37.0)	
3	16 (32.0)	18 (22.2)	
4	7 (14.0)	10 (12.3)	
5	0 (0.0)	2 (2.5)	

Values are presented as the mean ± standard deviation or number (%) as otherwise indicated. mTICI, modified treatment in cerebral ischemia; mRS, modified Rankin scale; NA, not applicable. *p-value calculated using Fisher’s exact test.

**Table 6:** Subgroup analysis of safety outcomes in embolic infarction by stent retriever diameter.

Variables	Solitaire 4 × 40 mm (*n =* 50)	Solitaire 6 × 40 mm (*n =* 81)	*p*
Vessel perforation	0 (0.0)	0 (0.0)	NA
Dissection	1 (2.0)	2 (2.5)	> 0.999^*^
Vasospasm	4 (8.0)	1 (1.2)	0.069^*^
Reocclusion	0 (0.0)	3 (3.7)	0.287^*^
Postprocedural hemorrhage	38 (76.0)	45 (55.6)	0.030
H-1	25 (50.0)	32 (39.5)	
H-2	11 (22.0)	7 (8.6)	
PH-1	2 (4.0)	3 (3.7)	
PH-2	0 (0.0)	3 (3.7)	
Symptomatic hemorrhage	2 (4.0)	4 (4.9)	> 0.999^*^

Values are presented as the mean ± standard deviation or number (%) as otherwise indicated. ICH, intracerebral hemorrhage; H, hemorrhagic infarction; PH, parenchymal hemorrhage; NA, not applicable. *p value calculated using Fisher’s exact test.

In this subgroup, the Solitaire 4 × 40 mm group had higher rates of embolization to new territory (26.0% vs. 7.4%, *p =* 0.007) and device switches (20.0% vs. 4.9%, *p =* 0.016), consistent with the overall cohort findings. The clinical outcomes at 3 months, as measured by mRS, were similar between the two groups among patients with embolic infarctions (2.32 ± 1.08 vs. 2.20 ± 1.20, *p =* 0.637).

Regarding safety outcomes, no significant differences were observed in vessel perforation or dissection rates between the groups. Vasospasm was more frequent in the Solitaire 4 × 40 mm group, albeit without significance (8.0% vs. 1.2%, *p =* 0.069). Postprocedural hemorrhage was significantly more common in the 4 × 40 mm group (*p* = 0.030), largely reflecting a numerical increase in H-1 and H-2 hemorrhages. However, the incidence of symptomatic hemorrhage was comparable between the groups (4.0% vs. 4.9%, *p* > 0.999).

### 3.6 Subgroup analysis of clinical and safety outcomes in M1 occlusion

A separate subgroup analysis was performed for patients with M1 segment occlusions to determine whether the efficacy and safety differences between the Solitaire 4 × 40 mm and 6 × 40 mm devices persisted in anatomically comparable occlusion sites (Tables 7, 8). The time from stroke onset to reperfusion and procedure time showed no significant differences between the two groups.

**Table 7:** Subgroup analysis of clinical and angiographic outcomes in M1 occlusion by stent retriever diameter.

Variables	Solitaire 4 × 40 mm (*n =* 54)	Solitaire 6 × 40 mm (*n =* 50)	*p*
Stroke onset to reperfusion (min)	294.85 ± 258.54	339.96 ± 236.40	0.355
Procedure time (min)	31.72 ± 18.17	27.98 ± 22.94	0.361
First-pass mTICI ≥2b	14 (25.9)	35 (70.0)	<0.001
First-pass mTICI 3	0 (0.0)	0 (0.0)	NA
No. of device passes	2.46 ± 1.13	1.48 ± 0.93	<0.001
>3 passes	0 (0.0)	0 (0.0)	NA
Angiographic outcome			
Final mTICI			0.033
3	36 (66.7)	46 (92.0)	
2b	15 (27.8)	4 (8.0)	
2a	1 (1.9)	0 (0.0)	
1	1 (1.9)	0 (0.0)	
0	1 (1.9)	0 (0.0)	
Final mTICI ≥2b	51 (94.4)	50 (100.0)	0.244^*^
Emboli to new territory	14 (25.9)	3 (6.0)	0.013
Device switch	7 (13.0)	1 (2.0)	0.061^*^
Rescue device used	6 (11.1)	2 (4.0)	0.273^*^
Reocclusion	3 (5.6)	4 (8.0)	0.708^*^
3-month mRS	2.28 ± 1.09	2.14 ± 1.14	0.550
0	2 (3.7)	4 (8.0)	
1	12 (22.2)	10 (20.0)	
2	17 (31.5)	18 (36.0)	
3	15 (27.8)	11 (22.0)	
4	8 (14.8)	7 (14.0)	
5	0 (0.0)	0 (0.0)	

Values are presented as the mean ± standard deviation or number (%) as otherwise indicated. M1, M1 segment of the middle cerebral artery; mTICI, modified treatment in cerebral ischemia; mRS, modified Rankin scale; NA, not applicable. *p-value calculated using Fisher’s exact test.

**Table 8:** Subgroup analysis of safety outcomes in M1 occlusion by stent retriever diameter.

Variables	Solitaire 4 × 40 mm (*n =* 54)	Solitaire 6 × 40 mm (*n =* 50)	*p*
Vessel perforation	0 (0.0)	0 (0.0)	NA
Dissection	1 (1.9)	1 (2.0)	> 0.999^*^
Vasospasm	5 (9.3)	0 (0.0)	0.057^*^
Reocclusion	3 (5.6)	4 (8.0)	0.708^*^
Postprocedural hemorrhage	41 (75.9)	25 (50.0)	0.011
H-1	27 (50.0)	20 (40.0)	
H-2	12 (22.2)	2 (4.0)	
PH-1	1 (1.9)	1 (2.0)	
PH-2	1 (1.9)	2 (4.0)	
Symptomatic hemorrhage	2 (3.7)	2 (4.0)	> 0.999^*^

Values are presented as the mean ± standard deviation or number (%) as otherwise indicated. *p value calculated using Fisher’s exact test. M1, M1 segment of the middle cerebral artery; ICH, intracerebral hemorrhage; H, hemorrhagic infarction; PH, parenchymal hemorrhage; NA, not applicable.

The Solitaire 6 × 40 mm group demonstrated superior first-pass efficacy, achieving a significantly higher rate of first-pass mTICI ≥2b reperfusion (70.0% vs. 25.9%, *p* < 0.001) and requiring fewer device passes (1.48 ± 0.93 vs. 2.46 ± 1.13, *p* < 0.001). The final angiographic outcomes also favored the 6 × 40 mm group, which achieved a significantly higher rate of complete reperfusion (mTICI 3: 92.0% vs. 66.7%, *p* = 0.033). Embolization to new territory occurred more frequently in the 4 × 40 mm group (25.9% vs. 6.0%, *p* = 0.013).

Regarding safety outcomes, postprocedural hemorrhage was significantly more common in the 4 × 40 mm group (75.9% vs. 50.0%, *p* = 0.011).

## 4 Discussion

In this multicenter study comparing Solitaire stent retrievers in patients with distal ICA and M1 occlusions, the 6 × 40 mm device demonstrated superior efficacy compared with the 4 × 40 mm device, while maintaining a comparable safety profile. The larger device achieved a significantly higher first-pass reperfusion rate and enhanced procedural efficiency, as evidenced by fewer device passes, a lower device switch rate, and reduced embolization to new territories. The improved efficacy was achieved without compromising safety outcomes. Although the 6 × 40 mm group had a lower postprocedural hemorrhage rate, the incidence of symptomatic hemorrhage was similar between the groups. These findings suggest that larger-diameter Solitaire stent retrievers may provide improved reperfusion rates and procedural efficiency in distal ICA and M1 occlusions, though device selection should remain individualized based on anatomical and clinical factors.

### 4.1 Impact of the stent retriever diameter on procedural efficacy

Our results clearly demonstrated the advantages of larger-diameter stent retrievers in treating distal ICA and M1 occlusions. The 6 × 40 mm device achieved significantly higher rates of first-pass mTICI ≥2b and mTICI 3 compared with the 4 × 40 mm device. The larger diameter may provide better wall apposition, particularly in larger vessels such as the ICA, thereby improving thrombus capture and removal (2). This enhanced clot capture capability could explain the reduced number of passes required for successful reperfusion and the lower device switching rate observed with the 6 × 40 mm device. Furthermore, the improved wall apposition potentially explains the lower rate of embolization to new territories observed with the 6 × 40 mm device.

Several prior investigations examining the correlation between the stent diameter and mechanical thrombectomy outcomes yielded conflicting results. Although Yi *et al* reported superior clinical outcomes with larger-diameter devices, subsequent studies reported varying results (8). Zaidat et al. (3) and Serna Candel et al. (6) found that stent length, rather than diameter, was the primary determinant of improved first-pass and overall reperfusion rates. Conversely, in patients with intracranial atherosclerotic stenosis, Yang et al. demonstrated superior outcomes with smaller-diameter stents (7). These contradictory findings might be attributable to the significant variability in stent retriever characteristics across studies. Previous research established that stent length directly influences treatment outcomes, with longer devices consistently achieving superior results (3–5). However, the heterogeneity of device parameters in existing studies—particularly varying lengths and product types—has limited our ability to isolate the specific impact of the stent diameter. The present study addressed this limitation by standardizing device length and product type. Furthermore, our multivariate analysis identified the use of the 6 × 40 mm stent retriever as an independent predictor of first-pass recanalization, providing robust evidence supporting the potential benefits of larger-diameter devices in mechanical thrombectomy.

### 4.2 Impact of stent retriever diameter on safety outcomes

In this study, we analyzed safety outcomes associated with different stent retriever diameters, specifically comparing the Solitaire 4 × 40 mm and 6 × 40 mm devices. The results demonstrated no significant difference in the rates of major safety events, such as vessel perforation, dissection, reocclusion, and symptomatic hemorrhage, between the two groups. However, the 4 × 40 mm device was associated with higher rates of vasospasm and postprocedural hemorrhage. Although these findings indicate a possible increase in specific adverse events with the smaller diameter, the differences were relatively minor, suggesting that both device sizes provide an acceptable safety profile in thrombectomy for acute ischemic stroke.

Safety outcomes related to the stent retriever diameter must be carefully considered, particularly regarding radial force. Previous studies demonstrated that excessive radial force can lead to adverse vessel wall outcomes such as endothelial injury and vessel wall trauma. Katz et al. documented histological evidence of intimal disruption and thickening resulting from high radial force during thrombectomy procedures (1). To mitigate the risk of vessel damage, it is essential to maintain an optimal and consistent radial force that balances clot capturing efficacy with vessel wall safety. Machi et al. demonstrated that constant radial force during retrieval is crucial for maintaining vessel wall apposition and stable clot engagement (2), with larger-diameter stent retrievers exhibiting superior performance concerning these characteristics.

Supporting this, manufacturer data revealed that although small-diameter stent retrievers such as the Solitaire 4 × 40 mm device deliver strong radial force in narrow vessels, their radial force diminishes with increasing vessel diameter (9). Conversely, the Solitaire 6 × 40 mm device maintains stable radial force across a wider range of vessel diameters, suggesting better adaptability to diverse vascular anatomies without compromising safety. Our study findings, which demonstrated comparable safety profiles between the devices but superior efficiency for the 6 × 40 mm device, support the safe implementation of larger-diameter stent retrievers in mechanical thrombectomy procedures.

### 4.3 Limitations

This study had some limitations. First, the retrospective design inherently introduced selection bias, leading to variability in both device selection and lesion distribution. Device selection (4 × 40 mm vs. 6 × 40 mm) was at the operator’s discretion and likely influenced by anatomical and clinical factors such as vessel diameter, clot burden, and procedural complexity. Notably, the 6-mm stent retriever was more frequently used in distal ICA occlusions, likely due to its ability to maintain stable radial force and ensure consistent vessel wall apposition, particularly in larger proximal vessels. In addition, distal ICA occlusions are often associated with a higher clot burden and a greater risk of distal embolic migration than M1 occlusions. Despite these procedural challenges, the 6 × 40 mm device demonstrated superior efficacy even under less favorable conditions, reinforcing its advantages and supporting the robustness of our findings.

Second, our outcome analysis was limited to the 3-month postprocedural period. Although this timepoint is widely accepted as the standard for assessing functional recovery in stroke trials, the lack of long-term follow-up data may limit our ability to detect delayed differences in clinical outcomes between the device groups.

Third, the generalizability of our findings was constrained by the exclusive evaluation of Solitaire devices. Thus, these results might not be fully applicable to stent retrievers with different mechanical properties. Additionally, although our findings primarily pertain to embolic occlusion in the anterior circulation (distal ICA and M1), their applicability to other stroke subtypes, particularly intracranial atherosclerotic stenosis (ICAS), requires careful consideration. ICAS presents unique challenges including distinct thrombus characteristics and complex vessel wall pathology and specific procedural considerations. Although our cohort included both embolic and ICAS cases, the predominance of embolic infarctions necessitates dedicated studies for ICAS-related occlusions.

Future investigations should address these limitations through prospective study designs that minimize selection bias and provide a more balanced representation of occlusion sites and device types. Incorporating diverse stent retriever types and systematically evaluating procedural decision-making criteria will be critical to understanding the factors influencing device selection. Additionally, focused analyses of specific stroke subtypes, such as embolic infarctions or ICAS, could provide deeper insights into the relationship between stent retriever diameter and outcomes. Such research would enhance our understanding of device performance across diverse anatomical and pathological contexts, ultimately informing evidence-based device selection strategies.

## 5 Conclusion

In this multicenter study, the Solitaire 6 × 40 mm stent retriever showed more favorable outcomes compared to the 4 × 40 mm device for distal ICA and M1 occlusions in our patient cohort. The 6 × 40 mm device was associated with higher reperfusion rates, fewer device passes, and lower rates of embolization to new territories, with comparable safety profiles. Our results suggest that the 6 × 40 mm stent retriever may be considered as a preferred option for distal ICA and M1 occlusions, though device selection should be individualized based on vessel anatomy, clot characteristics, and other clinical factors. While these findings primarily apply to embolic infarctions, further investigation is warranted for ICAS-related occlusions and broader patient populations to validate these observations.

## Data Availability

The raw data supporting the conclusions of this article will be made available by the authors, without undue reservation.
